# Development in a naturally acidified environment: Na^+^/H^+^-exchanger 3-based proton secretion leads to CO_2_ tolerance in cephalopod embryos

**DOI:** 10.1186/1742-9994-10-51

**Published:** 2013-08-29

**Authors:** Marian Y Hu, Jay-Ron Lee, Li-Yih Lin, Tin-Han Shih, Meike Stumpp, Mong-Fong Lee, Pung-Pung Hwang, Yung-Che Tseng

**Affiliations:** 1 Institute of Cellular and Organismic Biology, Academia Sinica, Taipei City, Taiwan; 2 Department of Life Science, National Taiwan Normal University, Taipei City, Taiwan; 3 Department of Aquaculture, National Penghu University of Science and Technology, Penghu, Taiwan

**Keywords:** V-type H^+^-ATPase, Rh proteins, Acid–base regulation, Embryonic development, Ocean acidification

## Abstract

**Background:**

Regulation of pH homeostasis is a central feature of all animals to cope with acid–base disturbances caused by respiratory CO_2_. Although a large body of knowledge is available for vertebrate and mammalian pH regulatory systems, the mechanisms of pH regulation in marine invertebrates remain largely unexplored.

**Results:**

We used squid (*Sepioteuthis lessoniana*), which are known as powerful acid–base regulators to investigate the pH regulatory machinery with a special focus on proton secretion pathways during environmental hypercapnia. We cloned a Rhesus protein (*slRhP*), V-type H^+^-ATPase (*slVHA*) and the Na^+^/H^+^ exchanger 3 (*slNHE3*) from *S. lessoniana*, which are hypothesized to represent key players in proton secretion pathways among different animal taxa. Specifically designed antibodies for *S. lessoniana* demonstrated the sub-cellular localization of NKA, VHA (basolateral) and NHE3 (apical) in epidermal ionocytes of early life stages. Gene expression analyses demonstrated that *slNHE3*, *slVHA* and *slRhP* are up regulated in response to environmental hypercapnia (pH 7.31; 0.46 kPa *p*CO_2_) in body and yolk tissues compared to control conditions (pH 8.1; 0.045 kPa *p*CO_2_). This observation is supported by H^+^ selective electrode measurements, which detected increased proton gradients in CO_2_ treated embryos. This compensatory proton secretion is EIPA sensitive and thus confirms the central role of NHE based proton secretion in cephalopods.

**Conclusion:**

The present work shows that in convergence to teleosts and mammalian pH regulatory systems, cephalopod early life stages have evolved a unique acid–base regulatory machinery located in epidermal ionocytes. Using cephalopod molluscs as an invertebrate model this work provides important insights regarding the unifying evolutionary principles of pH regulation in different animal taxa that enables them to cope with CO_2_ induced acid–base disturbances.

## Background

The embryonic development of most marine fish, crustaceans and molluscs takes place within an egg capsule that creates a barrier between the developing embryo and the surrounding environment to protect from biotic (e.g. predation) and abiotic stressors (e.g. osmo-protection) [1-4]. The opposite side of this protecting envelope is a limited diffusion permeability for gases such as O_2_ and CO_2_[5-8]. Diffusion coefficients of egg capsules from marine species, including cephalopods [5], sharks [5] and teleosts [9] usually range from 10 - 20% of pure seawater. As a consequence, embryos of oviparous organisms are exposed to high respiratory *p*CO_2_ and low *p*O_2_ within the egg capsule due to their increasing metabolic rate and the egg capsule wall acting as a diffusion barrier [5,10,11]. A recent study demonstrated that elevated environmental *p*CO_2_ is additive to the already high perivitelline fluid (PVF) *p*CO_2_ of cuttlefish eggs in order to maintain a sufficient diffusion gradient of CO_2_ (approximately 0.2 kPa) out of the egg [12]. Thus, elevated seawater *p*CO_2_ acts in an additive fashion on the naturally hypercapnic microenvironment within the egg, which may particularly challenge the acid–base regulatory machinery of the developing embryo.

Fish and cephalopods co-evolved during the Cambrian explosion around 550–530 million years ago [13,14] and are prime examples for convergent evolution shaped by the competition for similar resources in the marine environment [15,16]. The fact that fish [17,18] and cephalopod [12] embryos can tolerate seawater acidification (up to pH 7.3; leading to a pH of approximately 7.0 within the egg capsule), is believed to be associated with the presence of an efficient acid–base regulatory machinery. Recent studies demonstrated that in convergence to teleosts, cephalopods evolved epidermal ionocytes to mediate acid–base regulation before adult-type ion-regulatory epithelia (e.g. gills, kidneys and intestine) become fully functional [19-22]. In teleosts one type of ionocyte, the so called proton pump rich (HR) cells, which express V-type H^+^-ATPase (VHA), Na^+^/H^+^ exchangers (NHE), Rh glycoprotein c (Rhcg) and Na^+^/K^+^-ATPase (NKA), are believed to be specialized in the secretion of acid equivalents [20]. It is proposed that ammonia transporters from the Rh family in combination with NHE3, expressed in HR cells are key players in mediating the active secretion of ammonia and protons in seawater teleosts [23,24].

Although a previous study demonstrated that NHE3 expressing epidermal ionocytes of cephalopod embryos are also involved in active secretion of acid equivalents [22], there is no information regarding the existence and function of Rh proteins in cephalopod molluscs. Generally, information regarding the molecular characteristics and functionality of Rh proteins is very limited for marine invertebrates although many of them are ammonoteles, relying on efficient ammonia excretion pathways [25]. Rh proteins are of particular interest in the context of CO_2_-induced acid–base disturbances as they were proposed to have dual NH_3_/CO_2_ transport function [24,26,27]. Thus, Rh proteins may represent key players of acid–base regulation and gas exchange during environmental hypercapnia by facilitating CO_2_ diffusion.

The effects of elevated seawater CO_2_ partial pressure on marine organisms have recently moved into the focus of interest due to rising atmospheric CO_2_ concentrations which is expected to decrease the ocean surface pH by 0.5 units by the year 2100 and by 0.8 to 1.4 pH units by the year 2300 [28,29]. Besides the CO_2_ entry from the atmosphere, leakage of CO_2_ from sub-seabed carbon capture storage (CCS) sites may represent another potential anthropogenic source for local hypercapnia which could severely impact benthic biota [30,31]. CO_2_ induced reductions in seawater pH were shown to alter growth and development in early life stages of several marine invertebrate taxa, including echinoderms [32], crustaceans [33] and molluscs [12]. It has been hypothesized that the tolerance towards elevated water *p*CO_2_ is strongly related to the acid–base regulatory abilities of an organism [34]. However, the phenomenon of developmental delay observed for several marine taxa in response to seawater acidification has been suggested to be associated with energy allocation towards compensatory mechanisms like pH regulation and calcification [35-37].

The present work aims at identifying conserved acid–base regulatory mechanisms in marine invertebrates. Cephalopods are prime candidates for these studies due to their strong acid–base regulatory abilities despite their early phylogenetic position within the protostomes. In order to gain a mechanistic understanding of the pH regulatory machinery, immunocytochemical techniques will be applied to clarify the subcellular localization of acid–base relevant transporters located in epidermal ionocytes. We hypothesize that ion-transporters including NHE3 and V-type H^+^-ATPase constitute important components of the cephalopod pH regulatory machinery, which are essential to cope with CO_2_ induced acid–base disturbances. Additionally, the present work investigates the presence of Rh proteins in cephalopod molluscs, and their potential role in mediating pH homeostasis during environmental hypercapnia. A potential coupling of NH_3_ and H^+^ excretion/secretion is proposed, which may represent a fundamental pathway of pH regulation in ammonotelic organisms.

## Results

### Growth and perivitelline fluid abiotic parameters

**Figure 1:**
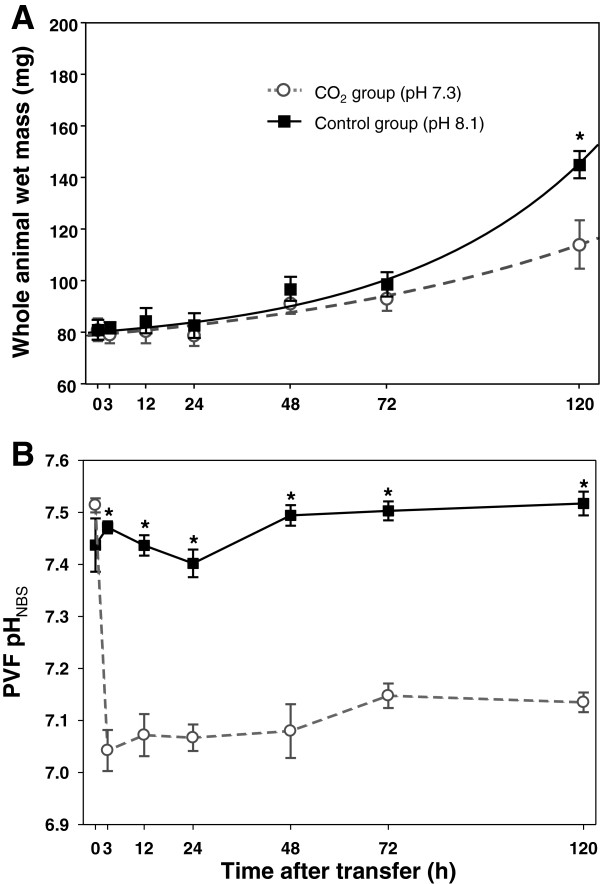
**Development and perivitelline fluid abiotic parameters during exposure to acidified conditions.** Effects of elevated ambient *p*CO_2_  on growth and development of *Sepioteuthis lessoniana* embryos **(A)**. Under control conditions wet mass (WM) increased during the incubation period following an exponential function (solid line) leading to a doubling of WM after the 120 h incubation period. Under elevated *p*CO_2_ conditions (dashed line) wet mass was significantly reduced after 120 h CO_2_ exposure. pH in the perivitelline fluid (PVF) of eggs exposed to control and acidified conditions **(B)**. Under control conditions PVF has a pH of 7.4 to 7.5 and decreases within 3 h of exposure to acidified conditions to a pH of 7.1. Asterisks indicate significant differences between treatments. Values are expressed as means ± SE (*n* = 3; with the average of three animals from each replicate).

To determine if elevated CO_2_ imposes an energetic cost to squid development and a stress to acid–base balance we first examined the developmental rates of the squid and PVF pH and *p*CO_2_ in response to elevated CO_2_. At all time points of tissue sampling for quantitative real-time PCR all *Sepioteuthis lessoniana* eggs contained viable and normally developed embryos. During the 120 h incubation period squid embryos reared under control conditions increased their wet mass from 80 mg_WM_ to 152 mg_WM_ following an exponential function (Figure 1A). Also CO_2_ treated animals appeared fully differentiated and organogenesis was complete. However, the wet mass (WM) of *S. lessoniana* late-stage embryos at the time point of 120 h was significantly reduced in 0.46 kPa CO_2_ treated animals (110.0 ± 11.3 mg_WM_), compared to control animals (151.5 ± 4.2 mg_WM_). No significant differences were observed for the earlier time points. The perivitelline fluid pH (pH_PVF_) in control animals, varied between pH 7.4 to pH 7.5 (0.2-0.3 kPa *p*CO_2_) at an ambient seawater pH of 8.1 (Figure 1B and Additional file 1: Figure S1). In contrast, within three hours after exposure to seawater pH 7.3, pH_PVF_ dropped to values of 7.05 pH_PVF_ units and stayed low (pH_PVF_ 7.05-7.1; 0.6-0.7 kPa *p*CO_2_) along the experimental duration of 120 h.

### Molecular cloning and phylogeny of squid Na^+^/H^+^-exchanger 3 and Rh-protein

**Figure 2:**
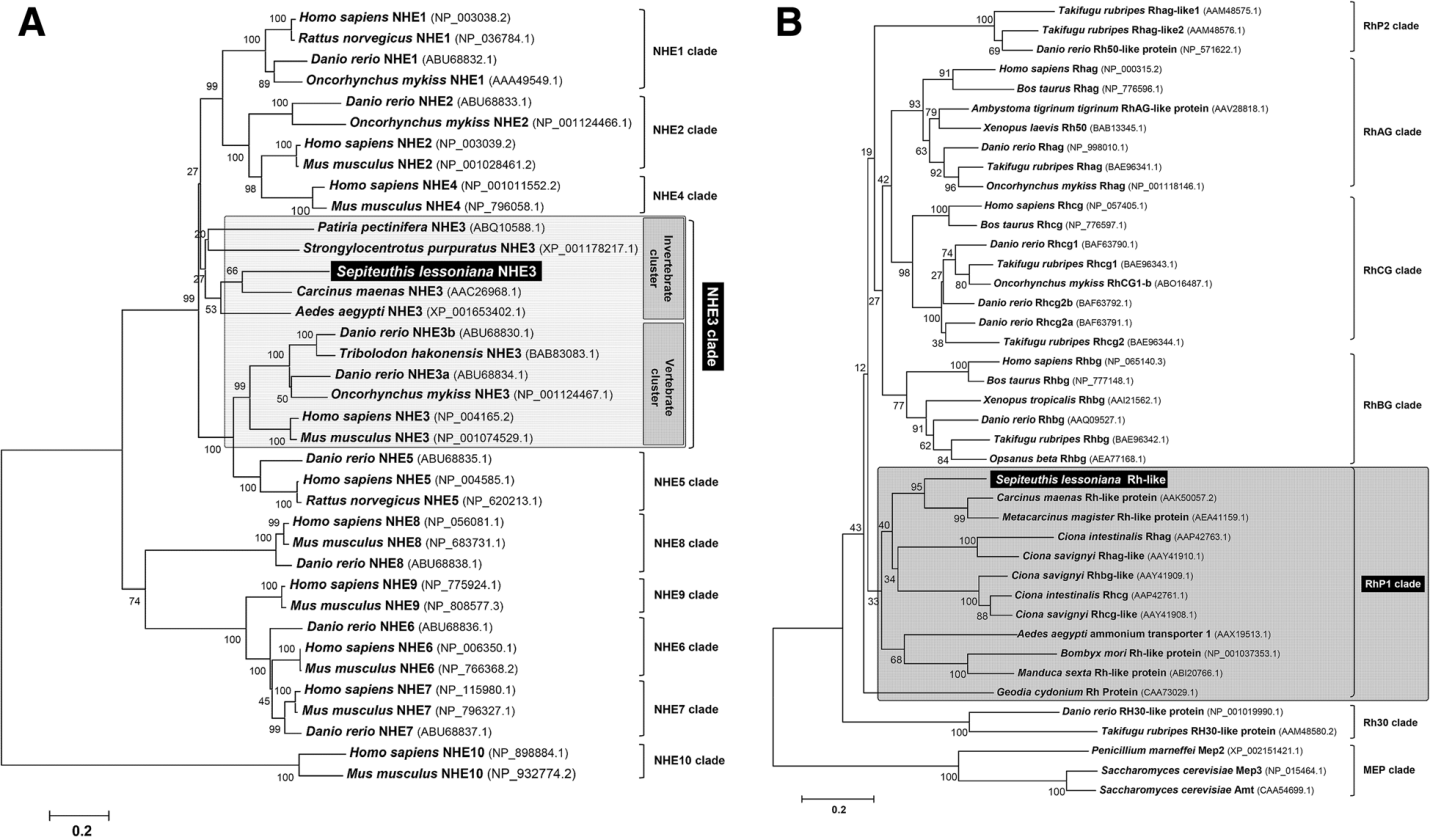
**Routed phylogenetic tree for *Sepioteuthis lessoniana *Na**^**+**^**/H**^**+**^**-exchanger isoform 3 (NHE3) (A) and Rh protein (RhP) (B).** Phylogenetic comparisons are based on amino acid sequences from NHE clades 1–10 as well as Rh isoforms, MEP and Amt as the out group. Numbers indicate bootstrap values and accession numbers for the sequences are provided along with species names.

Using RT-PCR followed by RACE technique we obtained partial (incomplete at the 5′end) cDNA sequences for NHE3 and Rh protein transcribed from *Sepioteuthis lessoniana* optic lobe mRNA. Translation of the predicted open reading frame resulted in a 533 and 264 amino acid long protein for slNHE3 and slRhP, respectively. Partial-length sequences of NHE isoforms and Rh proteins from different species were searched in the databases of NCBI or Ensembl and used for the phylogenetic analysis (Figure 2A + B). *S. lessoniana* NHE3 had highest degree of identity in amino acid sequence to orthologs of green shore crab *Carcinus maenas* (69%), and 31%, 23% and 21% identity in amino acid sequence to sea urchin, zebrafish and human NHE3, respectively. The phylogenetic analysis demonstrated that NHE3 divided into an invertebrate and a vertebrate cluster, with the invertebrate cluster being more similar to vertebrate NHE isoforms 1, 2 and 4 (Figure 2A and Additional file 2: Figure S2).

Squid RhP had highest degree of identity to the marine decapod crustaceans *Carcinus maenas* and *Metacarcinus magister* Rh-like proteins with 50% and 56% identity in amino acid sequence, respectively (Figure 2B and Additional file 2: Figure S2). This RhP1-clade consisting exclusively of invertebrates clearly separated from vertebrate RhBG, RhCG, RhAG clades as well as RhP2 and Rh30 clades.

### Tissue and ontogeny-dependent expression of acid–base transporters

**Figure 3:**
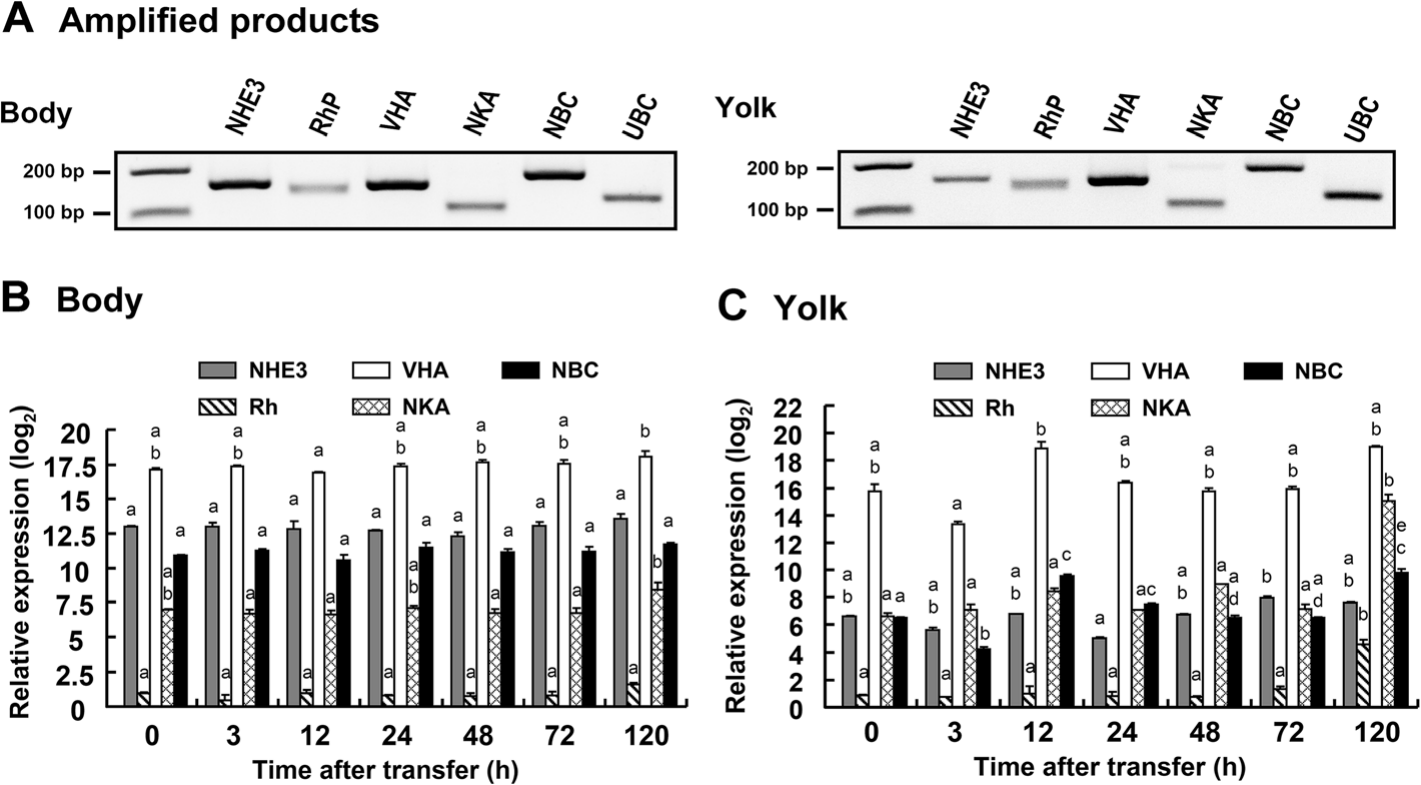
**Tissue specific expression levels of acid-base relevant genes.** Agarose gel of PCR-amplified cDNAs **(A)** for NHE3 (primer pair: slNHE3F1/slNHE3R1, predicted product size: 168), RhP (primer pair: slRhPF1/slRhPR1, predicted product size: 179) H^+^-ATPase (subunit A) (primer pair: slHAF1/slHAR1, predicted product size: 161), Na^+^/K^+^-ATPase (primer pair: slNKAF1/slNKAR1, predicted product size: 83), Na^+^/HCO_3_^-^ co-transporter (primer pair: slNBCF1/slNBCR1, predicted product size: 188) and the reference gene UBC (primer pair: slUCPF1/slUCPR1, predicted product size: 127). Relative expression of acid–base relevant genes in body **(B)** and yolk epithelium **(C)** homogenates along the period of 120 h. Different letters indicate differences between time points for each gene. Values represent mean (n = 3) ± SEM.

Using qPCR relative abundances of gene transcripts coding for acid–base relevant transporters including *slVHA*, *slNHE3*, *slNKA*, *slRhP* and *slNBCe* were determined in body tissues and the yolk epithelium, respectively (Figure 3A, B, C). In body tissues highest expression among acid–base transporters was found for *slVHA*, followed by *slNHE3*, *slNKA* and *slRhP*. In this tissue VHA expression was 1.3 log_2_-fold higher than *slNHE3*, 2.4 log_2_ fold higher than *slNKA* and 17 log_2_-fold higher than *slRhP* (Figure 3B). Along embryonic development within the experimental duration of 120 h (corresponding to developmental stage 23–27) expression of these genes remained relatively constant, with the exception of *slNKA* that increased mRNA levels in late stage embryos (120 h) by approximately 1.3 log_2_-fold (22%) (Figure 3B). Yolk tissues had a slightly different expression pattern compared to body tissues with highest expression levels for *slVHA*, followed by *slNHE3* and *slNKA* (that had similar levels of 7–9 log_2_-fold) and lowest expression levels of approximately 1 log_2_-fold were observed for *slRhP* (Figure 3C). In this tissue, *slVHA* expression was 2.1 log_2_-fold higher than *slNKA*, 2.4 log_2_ fold higher than *slNHE3* and 17 log_2_-fold higher than *slRhP*. Along the developmental period (120 h) *slNKA* and *slRhP* transcript levels in the yolk epithelium increased significantly after 120 h by 2.1 log_2_-fold (47%) and 4.25 log_2_-fold (78%) respectively (Figure 3C).

### Effects of seawater acidification on expression of acid–base transporters

**Figure 4:**
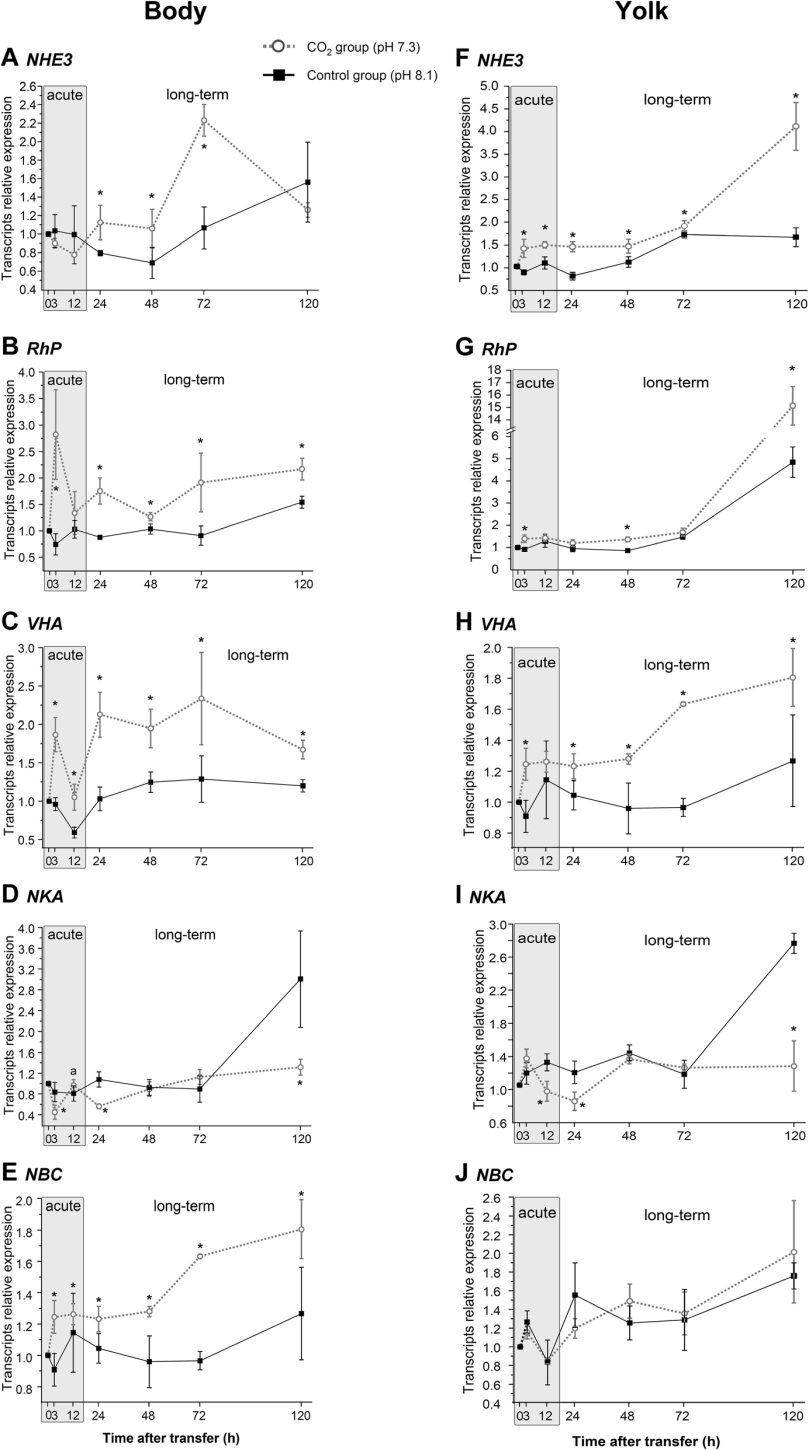
**Gene expression pattern during acclimation to acidified conditions.** Gene expression time series of acid–base relevant genes, including Na^+^/H^+^-exchanger 3 (*NHE3*) **(A)**, Rh protein (*RhP*) **(B)**, H^+^-ATPase (*VHA*) **(C)** Na^+^/K^+^-ATPase (*NKA*) **(D)** and the Na^+^/HCO_3_^-^ exchanger (NBC) **(E)** in homogenates of body (without yolk) and yolk tissues **(F**-**J)** exposed to control (solid line) and acidified (dashed line) conditions. Samples were taken at seven different time points along the incubation period of 120 h. Expression of the gene candidates are normalized to *slUBC*. Letters denote a significant difference between each time point whereas asterisks indicate significant differences between treatments. Values are expressed as means ± SE (*n* = 3).

Exposure to acidified seawater (pH7.3) at different time points along the incubation period of 120 h evoked differential and dynamic changes of acid–base transporter expression in body and yolk tissues during both, acute and long-term exposure (Figure 4A-H). During acute (12 h) exposure to low pH conditions no significant change has been observed for *slNHE3* and *slNKA* transcript abundance in body tissues (Figure 4A, D). Only during long-term exposure (24-72 h) *slNHE3* mRNA levels increased significantly by 36% (24 h to 48 h) and 52% (72 h), respectively. However, *slNHE3* transcript levels decreased back to control levels after 120 h exposure to pH 7.3. In contrast, *slRhP slVHA* and *slNBC* transcript abundances were immediately increased after three hours by 73%, 45% and 28% respectively (Figure 4B, C, E). Along the entire experimental period of 120 h *slRhP* and *slVHA* maintained significantly elevated expression levels in body tissues in response to acidified seawater.

Gene expression pattern of acid–base relevant genes on the yolk epithelium displayed similar general features as observed for body tissues. However, in contrast to body tissues, *slNHE3* immediately (within 3 h) increased and maintained transcript abundances by 29% to 13% above control levels in response to the low pH treatment (Figure 4F). Only at the last time point (120 h) expression of *slNHE3* and *slRhP* increased by 59% and 73%, respectively (Figure 4G + H). Also *slVHA* expressed in yolk epithelia increased expression in response to decreased seawater pH during both, acute and long-term exposure (Figure 4H). *slNKA* and *slNBC* mRNA levels remained unchanged or were significantly reduced in low pH treated animals at the time points 12 h, 24 h and 120 h (Figure 4I, J).

### Effects of seawater acidification on epidermal proton gradients

**Figure 5:**
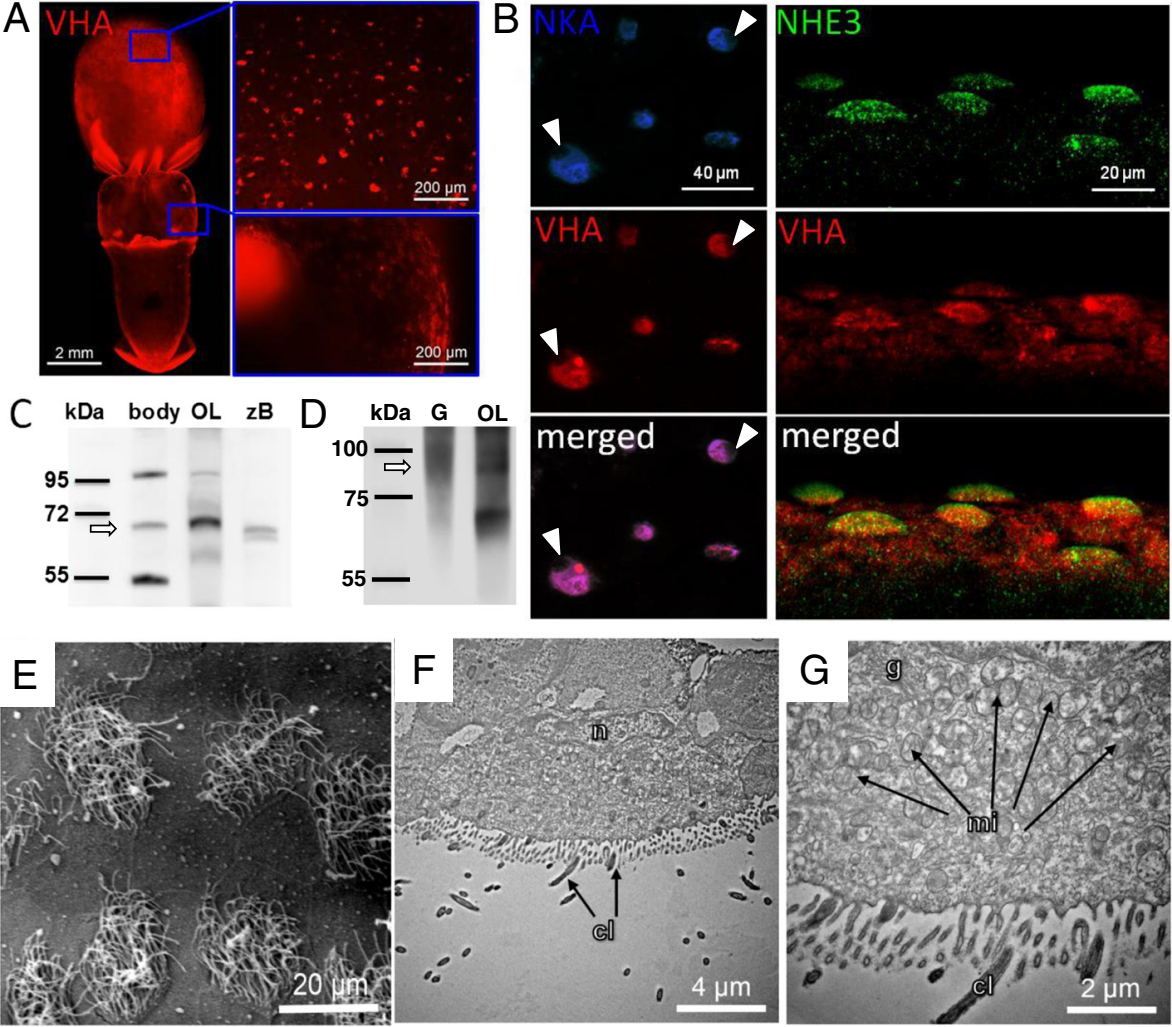
**Immunohistochemical and electron microscopical analyses of epidermal ionocytes.** Immunohistochemical detection of V-type H^+^-ATPase (VHA), Na^+^/K^+^-ATPase (NKA) and Na^+^/H^+^-exchanger 3 (NHE3) in epidermal ionocytes of squid *Sepioteuthis lessoniana*. VHA labeled ionocytes are distributed on yolk and head (overview image compiled from 11 images) **(A)**. VHA labeled ionocytes are scattered in a “salt and pepper” pattern mainly on the ventral side of the yolk and head. Confocal images of double immuno-fluorescent labeling of NKA indicates co-localization with VHA positive cells (left panel) **(B)**. VHA and NKA immuno reactivity demonstrates colocalization in epidermal ionocytes. Nuclei are covering the fluorescence signal (indicated by arrow head) suggesting a basolateral orientation of these transporters. Double labeling of VHA and NHE3 (right panel) confirms the basolateral orientation of VHA and demonstrates NHE3 localized in apical membranes (note the dome shaped apical surface of ionocytes labled by the NHE3 antibody). Western blot analysis of VHA subunit A in brain tissues of zebrafish (*Danio rerio*) (zB), optical lobe (OL), gills (G) and whole embryos (body) of squid (*Sepioteuthis lessoniana*), demonstrating immunoreactivity of the VHA antibody with a 70 kDa protein **(C)**. Western blot analysis of NHE3 in brain, optical lobe (OL) and whole embryo (body) homogenates, demonstrating immunoreactivity of the NHE3 antibody with a 95 kDa protein **(D)** (bands indicated by arrows). Scanning and transmission electron microscopy of ciliated epidermal ionocytes of squid (*S. lessoniana*) embryos **(E**-**G)**. Transmission electron microscopic images of surface epithelia demonstrating the ultrastuctural morphology of epidermal ionocytes in the head region which are particularly rich in mitochondria **(F**-**G)**. cilia (cl), golgi (g), mitochondrion (mi), nucleus (n).

Using H^+^-selective electrodes we determined proton gradients at different locations on the surface of squid embryos (stage 26–27) exposed to control and acidified conditions for 120 h (Figure 5A). Under control conditions, shallower proton gradients were detected on the yolk with a Δ[H^+^] of approximately 1.9 mmol over the entire yolk epithelium (points 1 to 3). On the skin close to the eye and the head (point 4 to 5) proton secretion had maximum Δ[H^+^] with up to 3.5 to 5 mmol, whereas on the mantle (point 6) proton Δ[H^+^] decreased again down to 0.6 mmol (Figure 5B). In low pH treated larvae [H^+^] gradients in the head region and the mantle were not significantly different compared to control animals. However, proton gradients measured on the yolk epithelium significantly increased by almost 2.5 mmol (56%) compared to control animals leading to comparable [H^+^] gradients as measured for points 4 and 5 in the head region (Figure 5B). The low pH induced increase in proton gradients on the yolk epithelium was depressed by 38 to 67% in the presence of 1 μM EIPA (Figure 5C). Also in the head region (locations 4 + 5) proton gradients were significantly depressed by 30–40% in response to 1 μM EIPA.

### Immunocytochemistry and ionocyte ultrastructure

**Figure 6:**
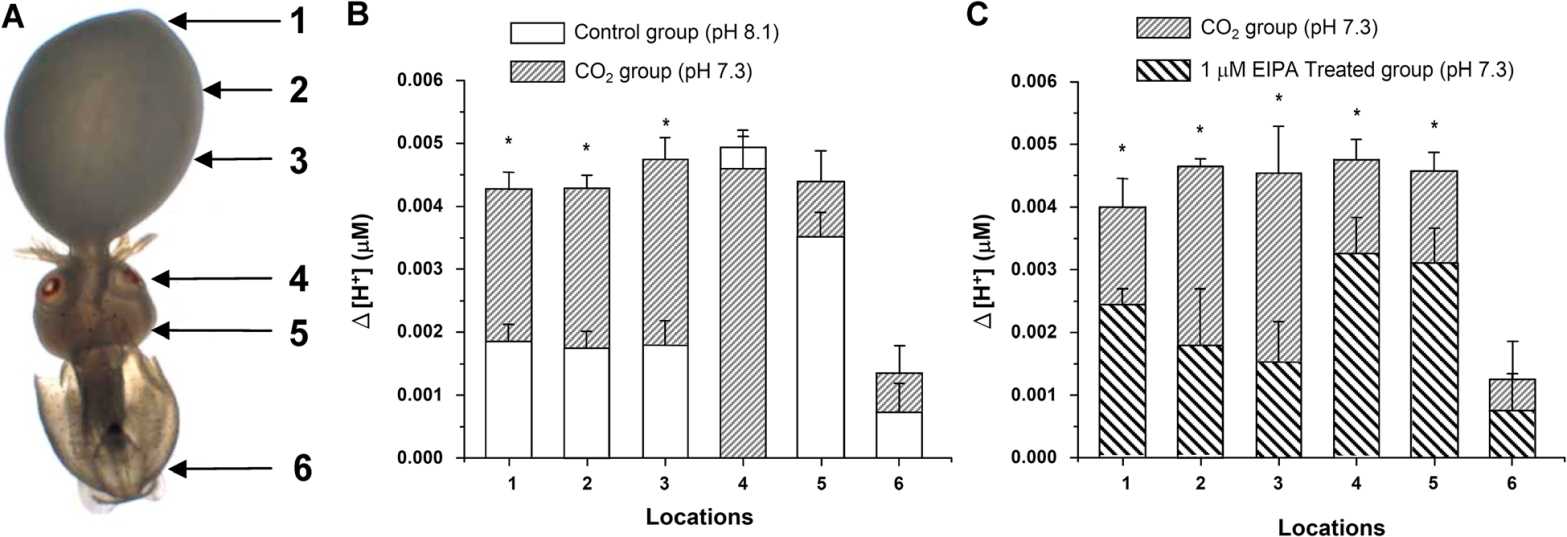
**Measurement of epidermal proton gradients.** Proton gradients were recorded on 6 spots along the lateral side of control and CO_2_ acclimated embryos **(A** + **B)**. Ion selective electrode technique measurements demonstrate that under control conditions the entire animal including yolk, head and mantle has positive ∆[H^+^] values with highest levels detected in the head region (spot 4 and 5) **(B)**. Lower H^+^ secretion values were recorded for the entire yolk surface. When exposed to elevated seawater *p*CO_2_ proton secretion increases significantly on the yolk. Increases in H^+^ gradients on the yolk, evoked by CO_2_ stimulation are sensitive to 1 μM EIPA **(C)**. Asterisks indicate significant differences between treatments (p < 0.02). Bars represent mean ± SE; (n = 3; with the average of 1–2 animals from each replicate).

Whole mount immunohistochemical analysis demonstrated that epidermal ionocytes which are scattered on the yolk sac epithelium and skin of squid embryos show a positive V-type-H^+^-ATPase (VHA) immunoreactivity using antibody specifically designed for *S. lessoniana* (Figure 6A). These VHA-rich cells are approximately 20 μm in diameter and occur in densities of 40–60 cells per 1 mm^2^. Double staining of NKA and VHA demonstrates that VHA is co-localized with Na^+^/K^+^-ATPase rich cells (NaRs) (Figure 5B; left panel). Nuclei are visible as dark spots in positively labeled ionocytes suggesting a basolateral localization of NKA and VHA. If immunoractivity would be located in apical membranes (above the nucleus) nuclei would not be visible. NHE3 and VHA double staining at a slope in the head region demonstrated that NHE3 is localized in dome-shaped apical membranes of epidermal ionocytes, and confirms the basolateral and potentially also cytosolic location of VHA (Figure 5B; right panel). Western blot analyses validate our VHA and NHE3 antibodies by recognizing a 70 kDa protein which is in the size range reported for VHA subunit A proteins from other species (Figure 5C) [38] as well as an about 95 kDa protein which is in the size range reported for NHE3 proteins from other species [39] (Figure 5D). In contrast to the extracts from the optical lobe (OL) where only one major band appears at 70 kDa two other bands at 100 and 55 kDa were visible in whole animal (body without yolk) extracts.

Additionally scanning electron microscopy was used to investigate ultrastructural features of ciliated epidermal ionocytes of developing squid embryos (Figure 5E-G). These images demonstrate that ciliated epidermal ionocytes are rich in mitochondria and thus, can be referred to as mitochondria rich cells (MRCs). MRCs in the head region are approximately 15–20 μm in diameter and 8–10 μm in height (Figure 5F) and have particularly high concentrations of mitochondria located close to the basolateral membrane (Figure 5F + G).

## Discussion

### Growth and development

Embryonic stages of the oval squid *Sepioteuthis lessoniana* respond with reduced growth and development towards decreased seawater pH along the time course of 120 h. This observation corroborates with earlier findings that demonstrated reduced developmental rates in response to chronic (6 weeks) exposure to 0.4 kPa (pH 7.3) seawater *p*CO_2_ in cuttlefish (*Sepia officinalis*) embryos [22]. The same study demonstrated that increases in seawater *p*CO_2_ are additive to the already high *p*CO_2_ (0.3 kPa; pH 7.4) within the egg leading to even higher *p*CO_2_ (0.7 kPa; pH 7.0) during exposure to 0.4 kPa seawater *p*CO_2_. As these observations were confirmed by the present work using squid (*S. lessonian*a) it can be concluded that an additive effect of environmental *p*CO_2_ seems to be a general feature in cephalopod egg masses in order to maintain a sufficient CO_2_ gradient between PVF and seawater.

A hypercapnia induced developmental delay as observed for several marine species including cephalopods, crustaceans, echinoderms, and bivalves [32,33,40-42] may be due to several reasons. It can be hypothesized that a hypercapnia induced developmental delay is caused by i) metabolic depression or ii) energy allocations or iii) a combination of both. An uncompensated acidosis has been suggested to induce metabolic depression, and thus, hypercapnia-born reductions in growth and development in aquatic organisms [43,44]. On the other hand, it was proposed that a higher fraction of energy is spent on acid–base regulation during environmental hypercapnia leading to less energy available for growth and developmental processes [32,37]. The present study demonstrated the up regulation of genes involved in primary- (e.g. V-type-H^+^-ATPases) and secondary-active (e.g. NHE3) acid-secretion pathways. This suggests that a higher fraction of energy is required to fuel the increased demands of the acid–base regulatory machinery of squid early life stages during exposure to acidified conditions. To test this hypothesis, future studies will confirm gene expression results by quantifying protein concentrations of acid–base transporters in response to seawater hypercania. Furthermore, determinations of metabolic rates in order to estimate acid–base regulatory costs during environmental hypercapnia represent an important task to fully understand the phenomenon of developmental delay during environmental hypercapnia.

### Acid–base regulation in cephalopod epithelia

In convergence to fish, cephalopods evolved ion regulatory epithelia in gills and integument, which are equipped with an ion regulatory machinery that is beneficial for coping with acid–base disturbances [12,22]. The present work demonstrates that gene transcripts including *Na*^*+*^*/H*^*+*^*exchanger 3 (NHE3)*, *V-type H*^*+*^*-ATPase (VHA)* and *Rh protein (RhP)* which are essential for proton equivalent secretion in vertebrates [23,45] are also expressed in cephalopod molluscs. Phylogenetic analysis of RhP and NHE3 sequences cloned from squid suggest that these proteins are conserved among invertebrates whereas more differentiation of isoforms and subtypes (e.g. Rhcg, Rhbg and Rhag) occurred during the evolution of vertebrates. Moreover, NKA-rich cells (NaRs) scattered on the yolk epithelium showed positive immunoreactivity for VHA and NHE3 using antibodies specifically designed for squid, *S. lessoniana*. These polyclonal antibodies were designed against conserved regions of the respective protein, and western blot analyses demonstrate specific immunoreactivity with proteins in the predicted size range. Depending on the tissue multiple bands were observed which may be due to immunoreactivity with VHA subunit A or fragments of the VHA associated glyco-proteins (mucous) or with proteins that occur in large quantities such as tubulin or actin. The cleanest signal with one major or one single band was achieved with samples from optical lobes and brain which have low amounts of connective tissues and which are rich in ion-transporters. Together with our previous work [22] it can be suggested that in this species there is only one type of ionocytes, which is characterized by high concentrations of VHA and NKA located in basolateral membranes, and with NHE3 being located in apical membranes. Finally, ultrastructural analyses of epidermal ionocytes demonstrate that these cells, particularly in the head and yolk region are rich in mitochondria, and thus can be referred to as mitochondria-rich cells (MRCs). These features resemble of proton pump rich (HR) cells described for brackishwater teleosts (*Oryzias latipes*) [46]. Interestingly, cephalopod ionocytes seem to have a basolateral orientation of the VHA. This feature has been described for base secreting type B intercalated cells in the mammalian kidney which are characterized by pendrin on the apical membrane and the absence of the AE-1 Cl^-^/HCO_3_^-^ exchanger on the basolateral side [47]. Also in euryhaline fish basolateral localization of the VHA has been associated with base secretory processes [48,49]. Although information is scarce for the pH regulatory systems of invertebrates a recent study suggested the interplay of VHA, NKA, NHE and carbonic anhydrase in the NH_3_/NH_4_^+^ secretion mechanism of the freshwater planarian, *Schmidtea mediterranea*[50]. Furthermore, in the green shore crab *Carcinus maenas* proton equivalent NH_3_/NH_4_^+^ secretion has been hypothesized to be achieved via trapping of NH_4_^+^ within VHA-rich vesicles and their subsequent exocytosis across the apical membrane [51]. These reports, together with the findings of the present work, demonstrate that further comparative studies are needed in order to gain knowledge regarding the differential function of VHA in pH regulatory systems of different animal taxa.

The fact that cephalopods were described as weak osmoregulators [52,53] but strong acid–base regulators [54,55] suggests, that epidermal ionocytes are mainly involved in acid–base regulatory processes. Acid–base regulatory abilities can be considered an essential feature in all animals due to the natural confrontation with respiratory CO_2_ that can cause extra- and intra-cellular pH disturbances [55,56]. Moreover, species with an oviparous early development may particularly require a potent acid–base regulatory machinery due to progressively increasing hypercapnic condition within the egg capsule [10,12]. From an evolutionary point of view it is tempting to speculate that the evolution of acid–base regulatory mechanisms which are already well established in older taxonomic groups, could have created the basis for more complex ion-regulatory systems of vertebrates.

Gene expression analysis performed on whole embryos and yolk epithelia demonstrated that along the time course, *slNHE3*, *slVHA*, *slRhP* and *slNBC* transcript abundance increases significantly in response to acidified conditions compared to control groups. This indicates that the acid–base regulatory machinery of squid early life stages responds to increased environmental hypercapnia by potentially increasing acid–base regulatory capacities. Although information from exclusively marine organisms is still scarce, few studies investigated the role of acid and ammonia extrusion mechanisms in euryhaline teleosts and crustaceans in response to elevated environmental *p*CO_2_ or ammonia concentrations [24,25,57-59]. Recently, a full length sequence for RhP from dungeness crab *Metacarcinus magister* was cloned and tested for transcript abundance during acclimation to elevated environmental ammonia levels [57]. This work indicated that at least during short-term (2 days) exposure, gill RhP together with VHA were significantly up-regulated in response to 1 mM NH_4_Cl suggesting a NH_4_^+^ trapping mechanism in the boundary layer of the gill surface [57]. Increased seawater CO_2_ concentrations comparable to those applied in the present work (0.3 kPa) induced increased ammonia secretion in the euryhaline shore crab *Carcinus maenas*[60]. However, in contrast to our findings expression levels of NHE and Rh-P in gill tissues of *C. maenas* were decreased or remained unchanged in response to the hypercapnia treatment. Furthermore, it was demonstrated for pufferfish *(Takifugu rubripes)* that in the seawater environment NHE3 in combination with apical Rhcg located in gill tissues are key players in the active secretion of ammonia and protons in response to 1 kPa CO_2_ induced hypercapnia [24]. In accordance to the findings of the present work a recent study conducted on a freshwater planarian (*Schmidtea mediterranea*) demonstrated that also in this early invertebrate, acidified conditions elicit increased ammonia excretion rates accompanied with higher expression of RhP and NKA [50]. Besides the secretion of proton equivalents several aquatic organisms, including fish [61] crustaceans [62] and cephalopods [54] were demonstrated to accumulate significant amounts of HCO_3_^-^ in order to buffer the excess of protons generated by the hydration of additional CO_2_ in body fluids. This process involves HCO_3_^-^ transporters in combination with cytosolic and membrane bound carbonic anhydrase to facilitate the reversible formation of HCO_3_^-^ from CO_2_[63,64]. Adult cuttlefish have been demonstrated to accumulate millimolar concentrations of HCO_3_^-^ in their blood in response to hypercapnia to partially stabilize pH_e_[54]. Although hemolymph HCO_3_^-^ measurements were not conducted in embryonic stages, mainly due to size limitations, the significant increases in *sINBCe* expression levels in body tissues suggest that HCO_3_^-^ buffering may also play an important role in acid–base regulation of early life stages.

The general mechanism of apical proton equivalent secretion via Rh proteins was described not only for teleost fish but also for the mammalian renal pH regulatory machinery [65]. Briefly, in kidney, pH balance is maintained by the basolateral HCO_3_^-^/Cl^-^ exchanger [66], apical V-type H^+^-ATPase and H^+^/K^+^-ATPase [47]. The secretion of protons into the lumen acidifies the urine [47] and shifts the equilibrium of NH_4_^+^/NH_3_ towards NH_4_^+^ and thereby trapping NH_4_^+^ in the luminal space resulting in net excretion of NH_4_^+^[65]. Within the cell, NH_4_^+^ is being deprotonated and NH_3_ is shuttled to the luminal side via Rhcg. However, in fish ionocytes it is proposed that the environmental acidification takes place within the micro environment (ca. 100 μm thickness) surrounding the surface of epidermal mitochondrion-rich cells [23]. The fact that in squid *slNHE3*, *slVHA* and *slRhP* were co-regulated in response to environmental hypercapnia, supports our hypothesis that these transporters are also key players in proton secretion pathways in this exclusively marine taxa. The involvement of NHE proteins in order to secrete acid equivalent displays an important pathway in the marine environment as it is thermodynamically favorable due to high external [Na^+^] (approximately 460 mM) compared to low intracellular [Na^+^] (approximately 30 mM) which provides a natural driving force [52,67]. As cephalopods are highly ammonotelic organisms, mainly excreting NH_3_/NH_4_^+^ it can be hypothesized that in cephalopods H^+^ and NH_3_/NH_4_^+^ secretion are coupled as well.

### Seawater acidification differentially affects acid–base transporters

In *Sepioteuthis lessoniana* embryos, two distinct hypercapnia acclimation phases were observed with respect to *slNHE3*, *slVHA* and *slRhP* mRNA expression levels in body tissues and the yolk epithelium. The first phase is an acute response (12 hours), and the second is a long-term acclimation phase (up to 120 h). During acute hypercapnia only *slRhP* and *slVHA* mRNA levels were immediately increased in body tissues whereas *slNHE3* expression was maintained at control levels. After long-term exposure *slNHE3* expression increased significantly, indicating an increased demand of this transporter. This observation suggests a biphasic acclimation mechanism at least in the integument of cephalopod embryos, which has also been described for various teleosts exposed to acute and chronic hypercapnia [68,69].

In the yolk epithelium increased expression levels of ion transporters, which are potentially involved in proton secretion mechanisms, corroborates with the increment of acid-secretion over the yolk surface determined by our SIET measurements. Gene expression analysis demonstrated more pronounced effects on the yolk epithelium compared to body (head and mantle) areas, which has been functionally supported by our SIET measurements, demonstrating significantly increased H^+^ gradients on the yolk epithelium as well. In yolk epithelia *slNHE3* and *slRhP* increased transcript abundance by 2.8 and 3.3 fold, respectively, whereas in body tissues no or only minor (1.8 fold) changes were detected for *slNHE3* and *slRhP* in response to 120 h exposure to hypercapnia. Together with our SIET measurements that demonstrated elevated, EIPA sensitive proton gradients on the yolk epithelium during hypercapnia it can be concluded that the yolk epithelium constitutes the major site of NHE3-dependent proton secretion. This displays a convergent feature to telosts, where the yolk epithelium functions as the major site of acid–base regulation during embryonic development before gills and kidneys become functional [19-22]. Interestingly, increased expression of *NBC* was only observed in body tissues, suggesting that HCO_3_^-^ homeostasis is mainly controlled via ion regulatory structures localized in body and head epithelia. The differential role of body vs. yolk epithelia in H^+^ secretion and HCO_3_^-^ accumulation mechanisms displays an important future task. In this context special attention will be dedicated to the potentially different role of bicarbonate transporters, carbonic anhydrase and the VHA in body vs. yolk ionocytes. Furthermore, the increased expression of RhP indicates that ammonia may represent a key pathway for the secretion of proton equivalents, which has already been proposed in earlier studies [23,58]. Recent studies demonstrated that increased NH_4_^+^ excretion rates seem to represent a common response in different marine taxa, including mollusks and echinoderms exposed to seawater acidification [35,37]. Thus, future studies are needed to address a potential coupling of proton and NH_3_/NH_4_^+^ secretion, which may represent a fundamental acid–base regulatory pathway in marine invertebrates.

## Conclusion

**Figure 7:**
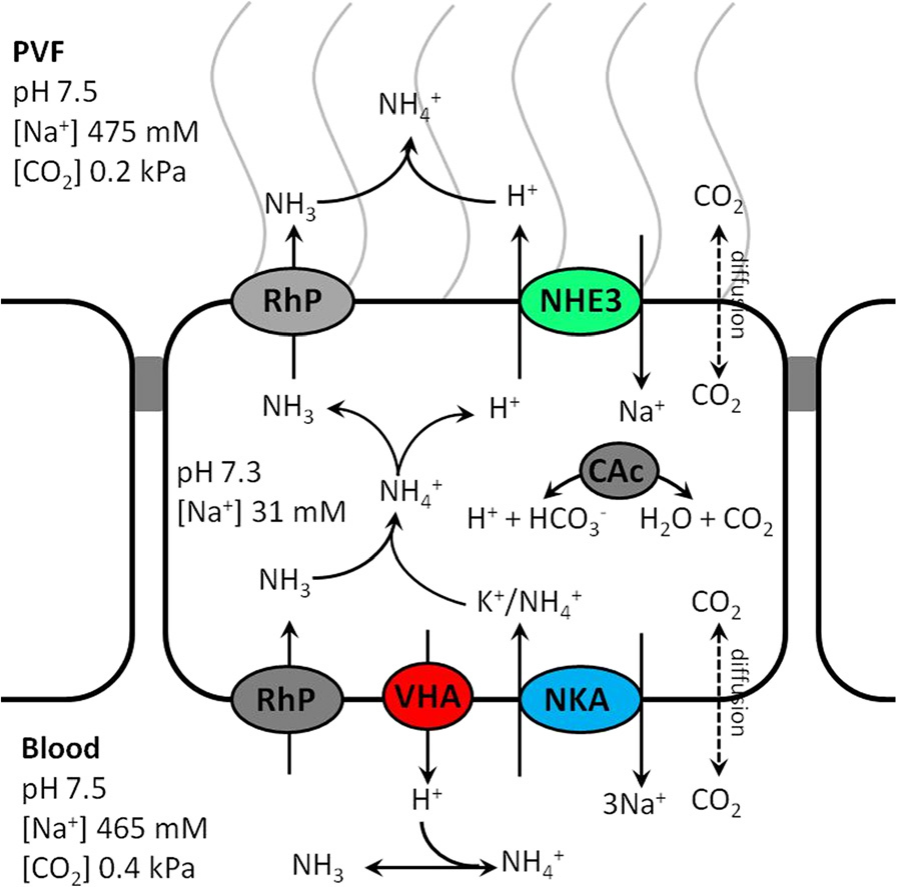
**Proposed mechanism of acid/ammonium regulation in the acid-secreting epidermal ionocyte of cephalopod embryos during exposure to seawater hypercapnia.** Confirmed transporters are colored whereas the sub-cellular localization of Rh proteins is not yet confirmed and thus hypothetical (grey). The net flux of respective substrates and diffusion through the membrane are indicated by arrows and numbers.

Squid, *Sepioteuthis lessoniana* is an excellent invertebrate model animal for studying the mechanisms of pH regulation in the marine environment. Cephalopod embryos possess an efficient acid–base regulatory machinery located in epidermal ionocytes which is summarized in the hypothetical model of Figure 7. These specialized cells express genes coding for proteins including NHE3, VHA and RhP which were identified as important players in vertebrate pH regulatory systems [65,70]. Especially the highly sensitive response of RhP expression during hypercapnic exposure can be of particular interest for future research as these proteins were suggested to have dual NH_3_ and CO_2_ transport function [71].

Future studies will use ion- and CO_2_-selective electrodes in combination with specific inhibitors in order to investigate acid–base regulatory mechanisms from a functional perspective. These studies will dedicate special attention to the mechanisms of NH_3_/NH_4_^+^ based proton secretion as well as the potential of Rh proteins to facilitate CO_2_ diffusion. This knowledge will lead to establishing a mechanistic basis of acid–base regulation in marine organisms, which will contribute to a general understanding of the unifying evolutionary principles of ion and acid–base regulation.

## Methods

### Experimental animals and CO_2_ perturbation experiment

*Sepioteuthis lessoniana* egg clusters were obtained from a local dealer on Peng-Hu island, Taiwan (ROC) in June 2011 and reared in a closed recirculating system (400 l total volume, nitrification filter, salinity 34.3 ± 0.06, temperature 25°C, constant 12 h dark:12 h light cycle) at the Institute of Cellular and Organismic Biology, Academia Sinica.

For the CO_2_ perturbation experiment, a group of 10–15 egg strands (stage 23; [72] were separately incubated in 5 l aquaria which were placed in a 300 l water bath, kept at a constant temperature of 25.6 ± 0.2°C. Along the incubation time of 120 h, we determined biometric parameters, perivitelline fluid (PVF) abiotic conditions and took tissue samples for expression analyses at seven different time points (0, 3, 12, 24, 48, 72 and 120 h). The nine tanks, with three replicate tanks for each treatment were randomly distributed within the water bath and connected to a flow through system providing natural seawater from a reservoir tank. In this reservoir tank the water was continuously pumped through a filter system and a UV disinfection unit and flow rates were adjusted to approximately 5 ml min^-1^ to guarantee high water quality inside the test aquaria. A light regime with a 12 h: 12 h light/dark-cycle was chosen. The aquaria were continuously equilibrated with the appropriate gas mixtures (8.13 and 7.31 pH) using a continuous pH-stat system (pH controller, MACRO) that controlled the addition of CO_2_ into the seawater, and aquaria were additionally continuously aerated with air (O_2_ saturation > 90%). Specific seawater conditions for the various incubations are given in Additional file 3: Table S1. Temperature, pH (NBS scale) and salinity were determined daily. pH_NBS_ was measured with a WTW 340i meter and WTW SenTix 81 electrode calibrated daily with Radiometer IUPAC precision pH_NBS_ buffers 7.00 and 10.00 (S11M44, S11 M007) to monitor the experiment and to adjust the pH-stat system. Additionally, water ammonia concentrations were determined every two to three days and levels were maintained < 0.1 mg l^–1^. Total dissolved inorganic carbon (*C*_T_) was measured in triplicate (100 μL each) using a Corning 965 carbon dioxide analyzer (Olympic Analytical Service (OAS), Malvern, U.K.). Seawater carbonate chemistry speciation was calculated from *C*_T_ and pH_NBS_ with the software CO2SYS [73] using the dissociation constants of Mehrbach et al. [74] as refitted by Dickson & Millero [75]. Embryos were staged according to Arnold [72]. For the determination of PVF pH, eggs were carefully lifted out of the water, and PVF (500 μl) was immediately sampled from the egg by using a syringe. A pH electrode (models WTW Mic and pH340i pH meter; WTW, Weilheim, Germany), calibrated before each measurement with Radiometer IUPAC precision pH_NBS_ buffers 7.00 and 10.00 (S11M44, S11 M007), was used to measure PVF pH in 1.5 ml Eppendorf tubes. During the measurement period (10–20 s) for pH, the PVF sample and sensors were placed in a thermostatted water bath at 25°C. For the sampling of embryos, egg strands were gently taken from the aquarium and the embryo was quickly (within 10 seconds) separated from the egg using a scissor and forceps. The embryo was weighted using a precision balance (Sartoris Extend) and body, as well as yolk tissues were immediately fixed in Trizol reagent (Invitrogen, Carlsbad, CA), homogenized (Tissue lyser, Quiagen, Hilden, Germany) and stored at -80°C. Samples for immunohistochemical analyses were fixed in 4% paraformaldehyde for 4 hours, and stored in 100% methanol. The experimental protocols were approved by the National Taiwan Normal University Institutional Animal Care and Utilization Committee (approval no.: 101005).

### Preparation of mRNA

Total RNA was extracted from the aqueous phase after addition of chloroform to Trizol homogenates and purified by addition of isopropanol. DNA contamination was removed with DNase I (Promega, Madison, WI, USA). The mRNA for the RT-PCR was obtained with a QuickPrep Micro mRNA Purification Kit (Amersham Pharmacia, Piscataway, NJ, USA) according to the supplier protocol. The amount of mRNA was determined by spectrophotometry (ND-2000, NanoDrop Technol, Wilmington, DE), and the mRNA quality was checked by running electrophoresis in RNA denatured gels. All mRNA pellets were stored at -20°C.

### Cloning of *slNHE3* and *slRhP* fragments and 5′ and 3′ rapid amplification of cDNA ends

Fragments of the *Sepioteuthis lessoniana Na*^*+*^*/H*^*+*^*-exchanger isoform 3 (slNHE3)* and Rh protein (*slRHP*) genes were isolated from optic lobe tissue by means of reverse transcription followed by PCR (RT–PCR) using degenerate primers designed for highly conserved regions. Reverse transcription was performed with Superscript RT (Invitrogen, Karlsruhe, Germany) and gene specific primers according to the manufacturer’s instructions with mRNA as templates. In the following PCR, primer pair 5′-GCTGTKGAYCCDGTKGCYGTBMTBGCHGT-3′ and 5′-TTVACYTTBAGCCAMTKHACCARNGGCTT-3′ resulted in a 233 bp fragment of the *NHE3* (BankIt1616044), the primer pair 5′-ATGRTCTTYGTGGGYTTCGGBTTYCTCATG-3′ and 5′-TTCTGGATRTGSACCATRTCMAVCTTSCCYTT-3′ resulted in a 195 bp fragment of *RhP* (BankIt1616407) cDNA was amplified using Ex Taq polymerase (Takara, Shiga, Japan) in the presence of 1.5 mM MgCl_2_ (PCR conditions: 3 min denaturation at 95°C, 35 sec annealing at 50°C and 30 sec elongation at 72°C, 40 cycles followed by a final amplification step of 7 min at 72°C). PCR fragments were separated after electrophoresis in 1.5% agarose gels. Extraction and purification of the PCR fragments from the gel was accomplished using the QIAquick^®^ gel extraction kit (QIAGEN, Hilden, Germany). Cloning and isolation of plasmids was performed using the pGEM^®^-T Easy Vector Systems and JM 109 competent *E. coli* cells (Promega, Madison, WI, USA). Plasmids were extracted using the Qiaprep Spin Miniprep Kit (QIAGEN, Hilden, Germany) and sequenced with an ABI 3700 sequencer (ABI, Warrington, UK). Sequence analysis was performed using the BLASTx program (NCBI, http://blast.ncbi.nlm.nih.gov/Blast.cgi). Specific primers of the 5′ and 3′ rapid amplification of cDNA ends (RACE) were designed from partial sequences obtained from the cloned PCR products. The RACE PCR program followed the manufacturer’s protocol, and RACE PCR products were also subcloned into the pGEM-T Easy vector and sequenced as described earlier.

### Real-time quantitative PCR (qPCR)

The mRNA expressions of target genes were measured by qPCR with the Roche LightCycler^®^ 480 System (Roche Applied Science, Mannheim, Germany). Primers for all genes were designed (Additional file 4: Table S2) using Primer Premier software (vers. 5.0; PREMIER Biosoft International, Palo Alto, CA). PCRs contained 40 ng of cDNA, 50 nM of each primer, and the LightCycler^®^ 480 SYBR Green I Master (Roche) in a final volume of 10 μl. All qPCR reactions were performed as follows: 1 cycle of 50°C for 2 min and 95°C for 10 min, followed by 45 cycles of 95°C for 15 sec and 60°C for 1 min (the standard annealing temperature of all primers). PCR products were subjected to a melting-curve analysis, and representative samples were electrophoresed to verify that only a single product was present (Figure 4A). All primer pairs used in this PCR had efficiencies >96%. Control reactions were conducted with nuclease-free water to determine levels of background. The standard curve of each gene was confirmed to be in a linear range with ubiquitin conjugated protein (*UBC*) as reference gene. The expression of this reference gene has been demonstrated to be stable in cephalopods among ontogenetic stages and during CO_2_ treatments [12].

### Scanning ion-selective electrode technique

SIET was used to measure H^+^ fluxes at the body surface of *Sepioteuthis lessoniana* embryos (stage 26–27) exposed to control ant low pH treatment. Glass capillary tubes (no. TW 150–4, World Precision Instruments, Sarasota, FL) were pulled on a Sutter P-97 Flaming Brown pipette puller (Sutter Instruments, San Rafael, CA) into micropipettes with tip diameters of 3–4 μm. These were then baked at 120°C overnight and vapor-silanized with dimethyl chlorosilane (Sigma- Aldrich) for 30 min. The micropipettes were backfilled with a 1 cm column of electrolytes and frontloaded with a 20 to 30 μm column of liquid ion exchanger cocktail (Sigma-Aldrich) to create an ion-selective microelectrode (probe). The following ionophore cocktail (and electrolytes) was used: H^+^ ionophore I cocktail B (40 mM KH_2_PO_4_ and 15 mM K_2_HPO_4_; pH 7). The details of the system were described previously [20]. To calibrate the ion-selective probe, the Nernstian property of each microelectrode was measured by placing the microelectrode in a series of seawater standard solutions (pH 7, 8 and 9). By plotting the voltage output of the probe against log [H^+^] values, a linear regression yielded a Nernstian slope of 57.59 ± 0.59 (*n* = 10) for H^+^.

### Measurement of surface H^+^ gradients

SIET was performed at room temperature (26°C) in a small recording chamber filled with 2 ml filtered natural seawater from the respective pH treatment (pH 8.1 and pH 7.3 corresponding to 0.057 kPa and 0.46 kPa CO_2_). Before the measurement, embryos were positioned in the center of the chamber with their dorsal side contacting the base of the chamber. The ion-selective probe was moved to the target position (10–20 μm away from the larval surface) to record the ionic activities, then the probe was moved away (10 mm) to record the background. Control and CO_2_ treated embryos were measured in an alternate mode, using seawater adjusted to the respective *p*CO_2_ as recording medium. Seawater pH in the recording chamber remained stable during the recording time (pH increase of acidified seawater < 0.5 pH units). The duration to measure one individual took around 10 min with 1–2 min for each reference and measuring point. After the recording, squid embryos were still alive and had normal mantle and heart contractions. Inhibitor experiments were performed as previously described [22] using the specific NHE inhibitor 5-ethylisopropyl amiloride (EIPA) (Sigma-Aldrich). EIPA was used to examine the inhibitory effects on increased proton secretion in *S. lessoniana* embryos exposed to hypercapnic conditions. EIPA was dissolved in DMSO (Sigma-Aldrich) and added to natural seawater at a final concentration of 1 μM. The final concentration of DMSO in working solution was 0.1% and did not affect proton transport [22]. In this study, a proton concentration gradient was determined by measuring H^+^ gradients between the two measuring points at the surface of larval skin and background.

### Immunohistochemical staining

Fixed samples were hydrated in a descending ethanol series and rinsed with PBS and incubated with 3% BSA for 30 min to block nonspecific binding. Samples were then incubated overnight at 4°C with the following primary antibodies: a Na^+^/K^+^-ATPase α1 of human origin (diluted 1:50; Na^+^/K^+^-ATPase α (H-300), Santa Cruz Biotechnology, INC.), which specifically recognizes the alpha subunit of the cephalopod NKA [76] as well as two polyclonal antibodies, one designed against synthetic peptides corresponding to the subunit A region (SYSKYTRALDEFYDK) of squid (*Sepioteuthis lessoniana*) V-type H^+^-ATPase (VHA) and a second against part of the carboxyl-terminal region (IYRVRKVGYDEQFIMSY) of Na^+^/H^+^-exchanger 3 (NHE3). After being washed with PBS, samples were further incubated in goat anti rabbit IgG Alexa-Flour 488 or 568 (dilution 1:300) for one hour. To allow double-color immunofluorescence staining, one of the polyclonal antibodies was directly labeled with Alexa Fluor dyes using Zenon antibody labeling kits (Molecular Probes, Eugene, OR, USA). After rinsing in PBS (3×5 min), samples were examined with a fluorescent microscope (Zeiss Imager 1 M) with an appropriate filter set and a confocal microscope (Leica TCS-SP5), respectively.

### Immunoblotting

For immunoblotting, 10 μL of crude extracts from the respective tissues were used. Crude extracts were centrifuged for 30 min at 19000 g and 4°C and supernatant was used for further analyses. Proteins were fractionated by SDS-PAGE on 10% polyacrylamide gels, according to Lämmli [77], and transferred to PVDF membranes (Millipore), using a tank blotting system (Bio-Rad). Blots were pre-incubated for 1 h at room temperature in TBS-Tween buffer (TBS-T, 50 mM Tris -HCl, pH 7.4, 0.9% (wt/vol) NaCl, 0.1% (vol/vol) Tween20) containing 5% (wt/vol) nonfat skimmed milk powder. Blots were incubated with the primary antibody (see previous section) diluted 1:250 at 4°C overnight. After washing with TBS-T, blots were incubated for 2 h with horseradish conjugated goat anti-rabbit IgG antibody (diluted 1:2,000, at room temperature; Amersham Pharmacia Biotech). Protein signals were visualized by using the enhanced chemiluminescence system (ECL, Amersham Pharmacia Biotech) and recorded using Biospectrum 600 imaging system (UVP, Upland, CA, USA).

### Electron microscopy

For scanning electron microscopy (SEM) observations, living squid embryos were removed from the egg capsule and immediately fixed in 4% paraformaldehyde with 5% glutaraldehyde for 10 h. Afterwards, the samples were transferred to a 0.1 M sodium cacodylate buffer solution and washed three times. The membrane fixation was performed with 1% OsO_4_ in 0.1 M PB for 30 min under a fume hood. After fixation the samples were washed in 0.1 M sodium cacodylate buffer. For dehydration the samples passed an ascending concentration of ethanol (50%, 70%, 80%, 95% and 100%). The samples were dried in a critical point drier (Hitachi HCP-2 CPD), gold coated (Cressington Sputter Coater 108), and observed using a scanning electron microscope (ESEM, FEI Quanta 200).

For TEM analyses, tissues were fixed and dehydrated as described for SEM analyses, then infiltrated and embedded in LR-white resin. Sections were cut with glass knives in a Leica EM UC7 ultramicrotome (Leica Microsystems GmbH, Wetzlar, Germany). Ultrathin sections (80 nm) were mounted on formvar-coated copper grids, double stained in uranyl acetate and lead citrate and examined with a Hitachi H-7000 electron microscope (Hitachi, Tokyo, Japan).

### Statistical analysis

Perivitelline pH, gene expression and inhibitor results of control and CO_2_ treated animals were analyzed using Student’s t-test using Sigma Stat 10.0 (Systat Software). One-way ANOVA followed by Tukey’s post-hoc test was used to compare expression levels of each gene along the experimental period of 120 h. Values are presented as means ± SE. Statistical analysis of qRT-PCR results was performed on mRNA quantities normalized to the housekeeping gene UBC. *Indicates a significant difference between treatment group and the respective control group (p < 0.05).

## Competing interests

The authors declare that they have no competing interests.

## Authors’ contributions

MYH, YCT and MS designed and conducted experiment, analyzed the data and compiled the manuscript. JRL conducted immunohistochemical experiments and analyzed the data. PPH designed the study and wrote the manuscript. LYL and THS conducted electrophysiological experiments and analyzed the data. MFL helped to conduct CO2 perturbation experiments and writing the manuscript. All authors read and approved the final manuscript.

## Supplementary Material

Additional file 1: Figure S1 Abiotic conditions of the perivitelline fluid (PVF) along the time course of the CO2 perturbation experiment. Within few hours of high pCO2 exposure PVF pCO2 increases, leading to an additive effect of environmental hypercapnia on PVF pCO2 levels (A). Total dissolved inorganic carbon (CT) in the PVF along the incubation period of 120 h. Values are presented as mean ± SEM (n = 3). Click here for file

Additional file 2: Figure S2 Multiple alignment of deduced NHE3 (A) and RhP (B) amino acid sequences in *Sepioteuthis lessoniana* (Accession numbers: NHE3 (BankIt1616044); Rh (BankIt1616407)) and *Carcinus maenas* (Accession numbers: NHE3 (AAC26968.1); Rh (AAK50057.2)). Residues in the consensus sequence are highlighted by a black background and represent absolutely conserved amino acids. Residues that are 80% or more conserved, and 62% or more conserved, are highlighted in dark and light gray, respectively. Click here for file

Additional file 3: Table S1 Primers used for qRT-PCR. Click here for file

Additional file 4: Table S2 Sea water physiochemical conditions in experimental setups along the incubation period of 120 h. Click here for file
