# Supplementary material for: Development in a naturally acidified environment: Na+/H+-exchanger 3-based proton secretion leads to CO2 tolerance in cephalopod embryos
Source: Front Zool. 2013 Aug 29;10:51. doi: 10.1186/1742-9994-10-51 (PMC3844404; doi:10.1186/1742-9994-10-51)
Supplement: Additional file 1: Figure S1 — Abiotic conditions of the perivitelline fluid (PVF) along the time course of the CO2 perturbation experiment. Within few hours of high pCO2 exposure PVF pCO2 increases, leading to an additive effect of environmental hypercapnia on PVF pCO2 levels (A). Total dissolved inorganic carbon (CT) in the PVF along the incubation period of 120 h. Values are presented as mean ± SEM (n = 3). [file 1742-9994-10-51-S1.pdf]

Abiotic conditions in the cephalopod egg

In addition to the perivitelline pH (PVF_{pH}) we determined total dissolved inorganic carbon (CT) from which CO_2 partial pressures were calculated. Briefly, in addition to pH samples another 500 µl of PVF was sampled with a gas-tight glass syringe for the determination of total dissolved inorganic carbon (*C*T). *C*T was measured in triplicate (100 µl each) using a Corning 965 carbon dioxide analyzer (Olympic Analytical Service, Malvern, UK). The carbonate system speciation was calculated from *C*T and pH NBS scale (pH_{NBS}) with the CO2SYS software (for details see the Material section of the main document).
The results demonstrate that environmental increases of pCO_2 increase PVF pCO_2 in an additive manner (Fig. S1 A).

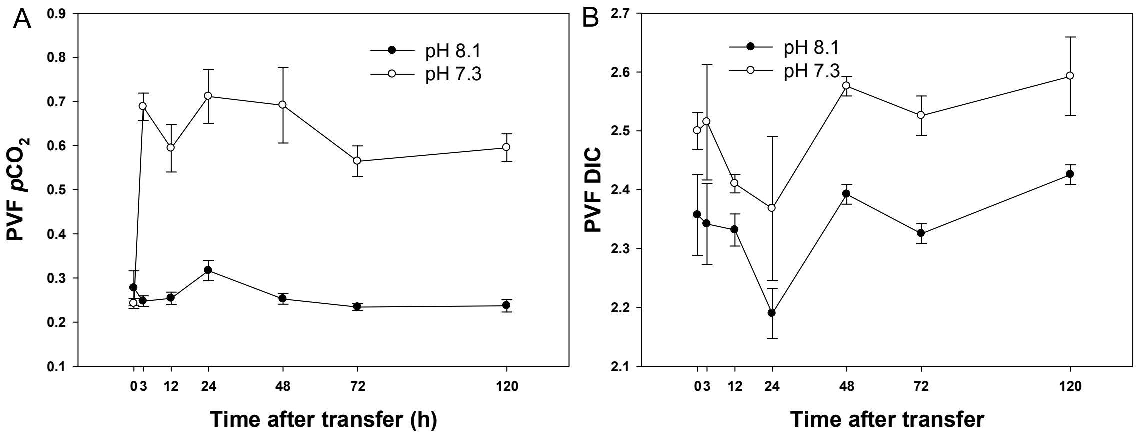

Figure S1 Abiotic conditions of the perivitelline fluid (PVF) along the time course of the CO_2 perturbation experiment. Within few hours of high pCO_2 exposure PVF pCO_2 increases, leading to an additive effect of environmental hypercapnia on PVF pCO_2 levels (A). Total dissolved inorganic carbon (CT) in the PVF along the incubation period of 120 h. Values are presented as mean± SEM (n=3).
