# Supplementary material for: Development in a naturally acidified environment: Na+/H+-exchanger 3-based proton secretion leads to CO2 tolerance in cephalopod embryos
Source: Front Zool. 2013 Aug 29;10:51. doi: 10.1186/1742-9994-10-51 (PMC3844404; doi:10.1186/1742-9994-10-51)
Supplement: Additional file 2: Figure S2 — Multiple alignment of deduced NHE3 (A) and RhP (B) amino acid sequences in Sepioteuthis lessoniana (Accession numbers: NHE3 (BankIt1616044); Rh (BankIt1616407)) and Carcinus maenas (Accession numbers: NHE3 (AAC26968.1); Rh (AAK50057.2)). Residues in the consensus sequence are highlighted by a black background and represent absolutely conserved amino acids. Residues that are 80% or more conserved, and 62% or more conserved, are highlighted in dark and light gray, respectively. [file 1742-9994-10-51-S2.pdf]

A. NHE3

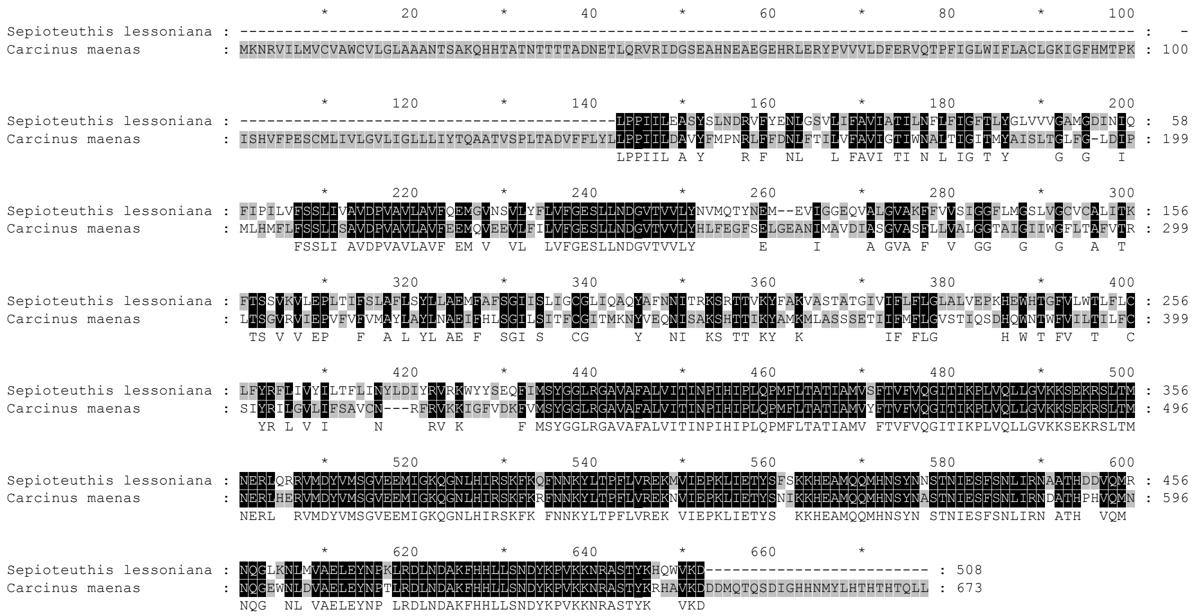

B. RhP

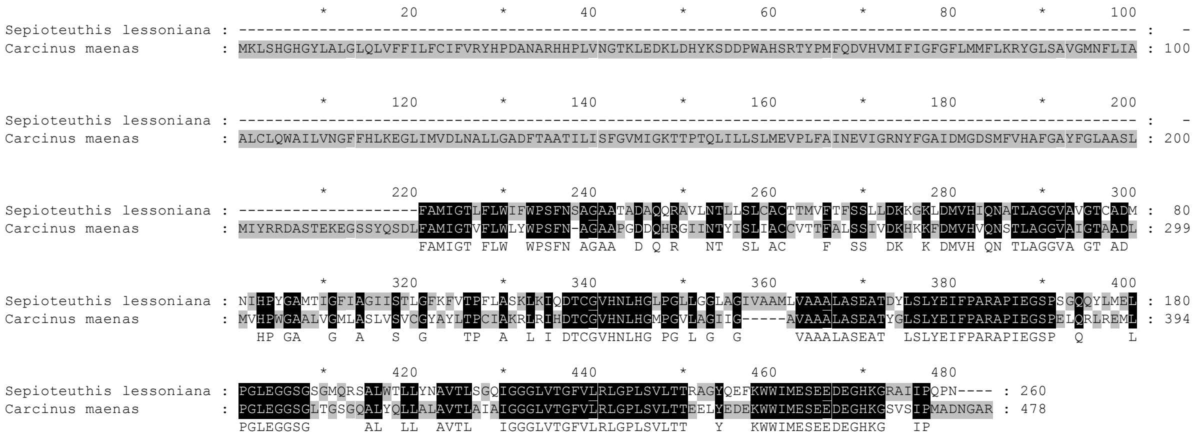

Figure S2. Multiple alignment of deduced NHE3 (A) and RhP (B) amino acid sequences in *Sepioteuthis lessoniana* (Accession numbers: NHE3 (BankIt1616044); Rh (BankIt1616407)) and *Carcinus maenas* (Accession numbers: NHE3 (AAC26968.1); Rh (AAK50057.2)). Residues in the consensus sequence are highlighted by a black background and represent absolutely conserved amino acids. Residues that are 80% or more conserved, and 62% or more conserved, are highlighted in dark and light gray, respectively.
