# Supplementary material for: Development in a naturally acidified environment: Na+/H+-exchanger 3-based proton secretion leads to CO2 tolerance in cephalopod embryos
Source: Front Zool. 2013 Aug 29;10:51. doi: 10.1186/1742-9994-10-51 (PMC3844404; doi:10.1186/1742-9994-10-51)
Supplement: Additional file 3: Table S1 — Primers used for qRT-PCR. [file 1742-9994-10-51-S3.pdf]

Table S1 Primers used for qRT-PCR

Gene name	Abbreviation	Primer sequence	Amplicon size (bp)	Accession numbers
Sodium–hydrogen exchanger 3	NHE3	F 5'- GGCTGTCTTCCAAGAAATGGGTGT -3'<br>R 5'- AAGAACTTGGCAACACCAAGAGCG -3'	168	BankIt1616044
Vacuolar-type H^+-ATPase	VHA	F 5'- ACGTGAGGGCAGTGTCAGTATTGT -3'<br>R 5'- TGATCAGCCAGTTGATGGAAGGGA -3'	161	ADM67602.1
Na^+, K^+-ATPase	NKA	F 5'- CCGTGCTGAATTTAAGGCAGGTCA -3'<br>R 5'- GCAAAGCTGATTCAGAAGCGTCAC -3'	83	GQ153672.1
Rhesus protein	RhP	F 5'-GCACAAAGGAAAGCTGGACATGGT-3'<br>R 5'-AATGATACCAGCCACCACTCCGA-3'	159	BankIt1616407
Sodium-bicarbonate cotransporter	NBC	F 5'-AATTCGCTGCATGATTGTCCGTCC-3'<br>R 5'-TTCCGGAGAACTGACGACCGATTT-3'	188	HM157263.1
Reference genes				
Cleavage and polyadenylation specificity factor	CPSF	F 5'- AAATCGCAGGTCGAGTGGATGAGT -3'<br>R 5'- TCGGTTCCATCACCTTGGACAACT -3'	130	HM157279.1
Ubiquitin-conjugated enzyme	UBC	F 5'- ATGCAGATGGCAGTATTTGCCTGG -3'<br>R 5'- TTATTGGCTGGGCTGTTTGGGTTC -3'	127	HM157280.1

F, forward primer; R, reverse primer
