# Supplementary material for: Development in a naturally acidified environment: Na+/H+-exchanger 3-based proton secretion leads to CO2 tolerance in cephalopod embryos
Source: Front Zool. 2013 Aug 29;10:51. doi: 10.1186/1742-9994-10-51 (PMC3844404; doi:10.1186/1742-9994-10-51)
Supplement: Additional file 4: Table S2 — Sea water physiochemical conditions in experimental setups along the incubation period of 120 h. [file 1742-9994-10-51-S4.pdf]

Table S2. Sea water physiochemical conditions in experimental setups along the incubation period of 120 h.

Parameter	Control	CO_2 treatment
**pH_{NBS}**	8.13±0.02	7.31±0.03
**pCO_2 (µatm)**	565±40	4561±76
**DIC (mM)**	2.47±0.02	2.81±0.09
**Salinity (‰)**	34.33±0.50	34.28±0.44
**Osmolarity (mOsmol/l)**	1044.33±5.60	1047.33±25.40
**Temperature °C**	25.62±0.21	25.61±0.20
**Ammonia (mg/l)**	<0.1	<0.1

Values are presented as means ± SD.
pCO_2, partial pressures of CO_2
